# Mortality of the Year

**Published:** 1867-01

**Authors:** 


### MORTALITY OF THE YEAR.

The following is the accurate, and complete statement of the number of deaths in each division of the City during the year, and their ages. The figures published in the *Tribune*, January 1st, for the month of December, was an estimate made, and varied from the official by only six. The total of deaths for that month were 309, instead of 315.

| MONTH. | NORTH DIV. | SOUTH DIV. | WEST DIV. | TOTAL. |
|---|---|---|---|---|
| January | 74 | 115 | 104 | 293 |
| February | 55 | 94 | 111 | 260 |
| March | 62 | 94 | 98 | 224 |
| April | 65 | 99 | 114 | 278 |
| May | 78 | 78 | 114 | 275 |
| June | 94 | 88 | 131 | 321 |
| July | 190 | 196 | 315 | 706 |
| August | 252 | 297 | 386 | 940 |
| September | 150 | 260 | 324 | 734 |
| October | 311 | 351 | 503 | 1,170 |
| November | 73 | 137 | 172 | 382 |
| December | 75 | 105 | 129 | 309 |
| Total | 1,479 | 1,904 | 2,501 | 5,926 |

The following is the table of ages:

| MONTHS. | Under Five. | Five to Twenty. | Twenty to Forty. | Forty to Fifty. | Fifty to Sixty. | Sixty to Eighty. | Over Eighty. | Unknown. |
|---|---|---|---|---|---|---|---|---|
| January | 152 | 29 | 55 | 17 | 14 | 18 | 1 | 7 |
| February | 117 | 40 | 52 | 20 | 9 | 11 | 3 | 8 |
| March | 122 | 25 | 62 | 12 | 7 | 17 | 0 | 9 |
| April | 129 | 33 | 59 | 24 | 11 | 14 | 1 | 7 |
| May | 134 | 25 | 58 | 22 | 7 | 17 | 2 | 10 |
| June | 165 | 36 | 54 | 21 | 14 | 14 | 0 | 15 |
| July | 518 | 46 | 80 | 22 | 12 | 18 | 2 | 8 |
| August | 578 | 80 | 156 | 48 | 27 | 32 | 3 | 16 |
| September | 329 | 84 | 183 | 68 | 41 | 25 | 3 | 7 |
| October | 329 | 161 | 392 | 116 | 72 | 69 | 6 | 25 |
| November | 173 | 45 | 75 | 40 | 15 | 23 | 3 | 8 |
| December | 153 | 34 | 62 | 16 | 11 | 23 | 6 | 4 |
| Total | 2,899 | 638 | 1,288 | 426 | 240 | 281 | 30 | 124 |

